# (4-Chloro­phen­yl)(3,6-dibromo-2-hy­droxy-7-meth­oxy-1-naphth­yl)methanone

**DOI:** 10.1107/S1600536810023299

**Published:** 2010-06-23

**Authors:** Ryosuke Mitsui, Atsushi Nagasawa, Shoji Watanabe, Akiko Okamoto, Noriyuki Yonezawa

**Affiliations:** a Department of Organic and Polymer Materials Chemistry, Tokyo University of Agriculture & Technology, 2-24-16 Naka-machi, Koganei, Tokyo 184-8588, Japan

## Abstract

The asymmetric unit of the title compound, C_18_H_11_Br_2_ClO_3_, contains two crystallographically independent mol­ecules in which the dihedral angles between the naphthalene ring systems and the benzene rings are 55.64 (11) and 60.50 (11)°. In each mol­ecule, an intra­molecular O—H⋯O=C hydrogen bond generates a six-membered ring. In the crystal structure, inter­molecular C—H⋯O and C—H⋯Cl hydrogen bonds and two different Br⋯O halogen bonds [2.9850 (19) and 3.2169 (19) Å] are observed.

## Related literature

For the structures of closely related compounds, see: Mitsui *et al.* (2008*a* ▶,*b* ▶, 2009 ▶, 2010*a* ▶,*b* ▶,*c* ▶). For a review of halogen bonding, see: Politzer *et al.* (2007 ▶).
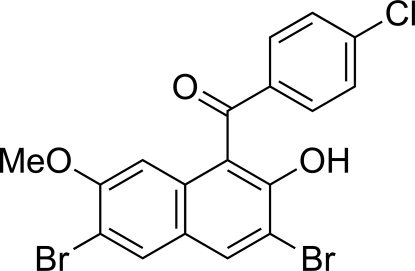


## Experimental

### 

#### Crystal data

C_18_H_11_Br_2_ClO_3_
*M* *_r_* = 470.54
Monoclinic, 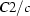

*a* = 32.1178 (6) Å
*b* = 11.1814 (2) Å
*c* = 19.7078 (4) Å
β = 104.687 (1)°
*V* = 6846.2 (2) Å^3^
*Z* = 16
Cu *K*α radiation
μ = 7.57 mm^−1^
*T* = 193 K
0.30 × 0.30 × 0.10 mm

#### Data collection

Rigaku R-AXIS RAPID diffractometer
Absorption correction: numerical (*NUMABS*; Higashi, 1999 ▶) *T*_min_ = 0.135, *T*_max_ = 0.469
60758 measured reflections
6263 independent reflections
5929 reflections with *I* > 2σ(*I*)
*R*_int_ = 0.036

#### Refinement

*R*[*F* ^2^ > 2σ(*F* ^2^)] = 0.031
*wR*(*F* ^2^) = 0.081
*S* = 1.14
6263 reflections
435 parameters
H-atom parameters constrained
Δρ_max_ = 0.85 e Å^−3^
Δρ_min_ = −1.08 e Å^−3^

### 

Data collection: *PROCESS-AUTO* (Rigaku, 1998 ▶); cell refinement: *PROCESS-AUTO*; data reduction: *CrystalStructure* (Rigaku/MSC, 2004 ▶); program(s) used to solve structure: *SIR2004* (Burla *et al.*, 2005 ▶); program(s) used to refine structure: *SHELXL97* (Sheldrick, 2008 ▶); molecular graphics: *ORTEPIII* (Burnett & Johnson, 1996 ▶); software used to prepare material for publication: *SHELXL97*.

## Supplementary Material

Crystal structure: contains datablocks I, global. DOI: 10.1107/S1600536810023299/ez2214sup1.cif

Structure factors: contains datablocks I. DOI: 10.1107/S1600536810023299/ez2214Isup2.hkl

Additional supplementary materials:  crystallographic information; 3D view; checkCIF report

## Figures and Tables

**Table 1:** Hydrogen-bond geometry (Å, °)

*D*—H⋯*A*	*D*—H	H⋯*A*	*D*⋯*A*	*D*—H⋯*A*
O2—H2*O*⋯O1	0.79	1.77	2.497 (3)	153
O5—H5*O*⋯O4	0.78	1.85	2.568 (3)	153
C4—H4⋯O4^i^	0.95	2.42	3.338 (3)	162
C18—H18*A*⋯Cl2^ii^	0.98	2.81	3.406 (3)	120
C34—H34⋯O2^iii^	0.95	2.49	3.397 (4)	160

Symmetry codes: (i) 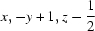; (ii) 
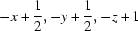
; (iii) 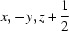.

**Fig. 1.**
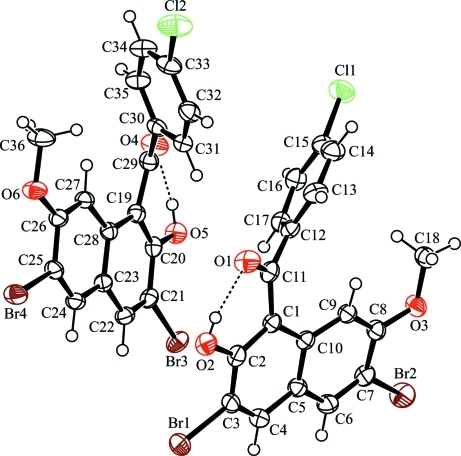
The asymmetric unit of compound (I), showing 50% probability displacement ellipsoids. The intramolecular hydrogen bond is shown as a dashed line.

**Fig. 2.**
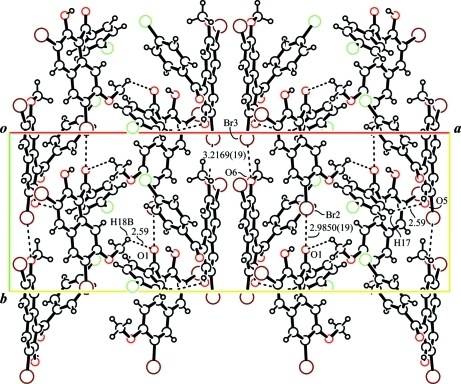
Partial crystal packing diagram of compound (I), viewed down the c axis. Intermolecular C—H···O hydrogen bonds and Br···O halogen bonds are shown as dashed lines.

**Fig. 3.**
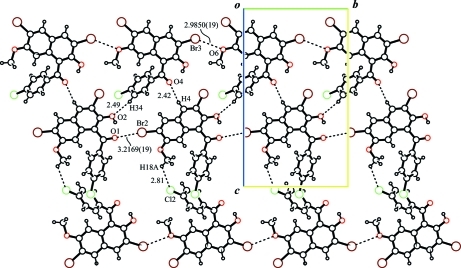
Partial crystal packing diagram of compound (I), viewed down the a axis. Intermolecular C—H···O, C—H···Cl hydrogen bonds and Br···O halogen bonds are shown as dashed lines.

## supplementary crystallographic information

### Comment

Recently, we reported the crystal structures of 1-aroylated 2,7-dimethoxynaphthalenes, 1-(4-chlorobenzoyl)-2,7-dimethoxynaphthalene (Mitsui *et al.*, 2008*a*), (4-chlorophenyl)(2-hydroxy-7-methoxynaphthalen-1-yl)methanone (Mitsui *et al.*, 2008*b*), (4-chlorophenyl)(2-ethoxy-7-methoxynaphthalen-1-yl)methanone (Mitsui *et al.*, 2009), 1-bromo-8-(4-chlorobenzoyl)-7-hydroxy-2-methoxynaphthalene (Mitsui *et al.*, 2010*a*), (8-bromo-2,7-dimethoxy-1-naphthyl)(4-chlorophenyl)methanone (Mitsui *et al.*, 2010*b*) and (4-chlorophenyl)(3,8-dibromo-2-hydroxy-7-methoxy-1-naphthyl)methanone (Mitsui *et al.*, 2010*c*). As a part of our ongoing studies on the synthesis and crystal structure analysis of aroylated naphthalene derivatives, we prepared and analysed the structure of a single crystal of 2,7-dibromo-4-(4-chlorobenzoyl)-3-hydroxy-6-methoxynaphthalene, (I). The title compound was prepared by electrophilic aromatic bromination reaction of (4-chlorophenyl)(2-hydroxy-7-methoxynaphthalen-1-yl)methanone with bromine.

An *ORTEPIII* (Burnett & Johnson, 1996) plot of (I) is shown in Fig. 1. The title compound crystallizes in the monoclinic crystal system such that there are two molecules in the asymmetric unit, molecules *A* and *B*, respectively. In *A*, the dihedral angle between the naphthalene ring (C1–C10) and the benzene ring (C12–C17) is 55.64 (11)°, and the central carbonyl C—(C═O)—C group is relatively coplanar to the naphthalene ring [9.72 (15)°]. In *B*, by contrast, the dihedral angle between the naphthalene ring (C19–C28) and the benzene ring (C30–C35) is 60.50 (11)°, and the central carbonyl C—(C═O)—C group is twisted away from the naphthalene ring [23.73 (15)°]. In each molecule, the hydroxy groups are involved in O—H···O═C hydrogen bond generates a six-membered ring (Fig. 1 and Table 1).

In the crystal structure, intermolecular C—H···O and C—H···Cl hydrogen bonding interactions contribute to the stabilization of the molecular and crystal structures (Figs. 2 and 3, Table 1). Additionally, the contact distances Br2···O1 and Br3···O6 are 2.9850 (19) and 3.2169 (19) Å, respectively (Figs. 2 and 3). These contacts are shorter than the sum of their van der Waals radii (3.37 Å), and arranged nearly linearly [C7—Br2···O1 = 172.24 (10)°, C21—Br3···O6 = 142.02 (9)°], suggesting that there is a possibility for halogen bonding, which further contributes to crystal packing stability (Politzer *et al.*, 2007).

### Experimental

To a solution of (4-chlorophenyl)(2-hydroxy-7-methoxynaphthalen-1-yl)methanone (313 mg, 1.00 mmol) in chloroform (5 ml) was added Br_2_ (646 mg, 4.04 mmol) drop-wise at 0 °C. The reaction mixture was stirred for 12 h at 0 °C, then poured into aqueous 2 *M* Na_2_S_2_O_3_ (10 ml), and the aqueous layer was extracted with CHCl_3_ (3 × 10 ml). The combined organic layers were washed with 2 *M* Na_2_S_2_O_3_ (3 × 30 ml) and brine (3 × 30 ml), and dried over MgSO_4_ overnight. The solvent was removed *in vacuo* and the crude material was purified by column chromatography (silica gel, CHCl_3_) to give the title compound (yield 409 mg, 87%). Single crystals suitable for X-ray diffraction analysis were obtained from CHCl_3_ as yellow blocks (m.p. 431.5–432.0 K).

Spectroscopic Data: ^1^H NMR (300 MHz, CDCl_3_) δ 10.10 (s, 1H), 8.04 (s, 1H), 7.88 (s, 1H), 7.63 (d, 2H), 7.43 (d, 2H), 6.62 (s, 1H), 3.49 (s, 3H); ^13^C NMR (75 MHz, DMSO-d_6_) δ 194.2, 154.0, 149.2, 138.4, 135.9, 132.3, 131.3, 130.6, 130.5, 128.6, 124.7, 121.0, 111.6, 110.8, 102.8, 55.7; IR (KBr): 1662, 1607, 1591, 1486, 1240, 1211, 1093, 843; HRMS (*m/z*): [*M* + H]^+^ calcd for C_18_H_12_Br_2_ClO_3_, 468.8842 found, 468.8839. Anal. Calcd for C_18_H_11_Br_2_ClO_3_: C 45.95, H 2.36. Found: C 46.10, H 2.32.

### Refinement

All the H atoms could be located in difference Fourier maps. All the H atoms were subsequently refined as riding atoms, with O2—H2O = 0.792, O5—H5O = 0.783, C—H = 0.950 (aromatic) and 0.980 (methyl) Å, and *U*_iso_(H) = 1.2*U*_eq_(C).

### Crystal data

**Table:**

C_18_H_11_Br_2_ClO_3_	*F*(000) = 3680
*M**_r_* = 470.54	*D*_x_ = 1.826 Mg m^−^^3^
Monoclinic, *C*2/*c*	Melting point = 431.5–432.0 K
Hall symbol: -C 2yc	Cu *K*α radiation, λ = 1.54187 Å
*a* = 32.1178 (6) Å	Cell parameters from 39111 reflections
*b* = 11.1814 (2) Å	θ = 3.2–68.2°
*c* = 19.7078 (4) Å	µ = 7.57 mm^−^^1^
β = 104.687 (1)°	*T* = 193 K
*V* = 6846.2 (2) Å^3^	Block, yellow
*Z* = 16	0.30 × 0.30 × 0.10 mm

### Data collection

**Table:**

Rigaku R-AXIS RAPID diffractometer	6263 independent reflections
Radiation source: rotating anode	5929 reflections with *I* > 2σ(*I*)
graphite	*R*_int_ = 0.036
Detector resolution: 10.00 pixels mm^-1^	θ_max_ = 68.2°, θ_min_ = 4.2°
ω scans	*h* = −38→38
Absorption correction: numerical (*NUMABS*; Higashi, 1999)	*k* = −13→13
*T*_min_ = 0.135, *T*_max_ = 0.469	*l* = −23→23
60758 measured reflections	

### Refinement

**Table:**

Refinement on *F*^2^	Primary atom site location: structure-invariant direct methods
Least-squares matrix: full	Secondary atom site location: difference Fourier map
*R*[*F*^2^ > 2σ(*F*^2^)] = 0.031	Hydrogen site location: difference Fourier map
*wR*(*F*^2^) = 0.081	H-atom parameters constrained
*S* = 1.14	*w* = 1/[σ^2^(*F*_o_^2^) + (0.0417*P*)^2^ + 12.8149*P*] where *P* = (*F*_o_^2^ + 2*F*_c_^2^)/3
6263 reflections	(Δ/σ)_max_ = 0.001
435 parameters	Δρ_max_ = 0.85 e Å^−^^3^
0 restraints	Δρ_min_ = −1.08 e Å^−^^3^

### Special details

**Table:**

Geometry. All e.s.d.'s (except the e.s.d. in the dihedral angle between two l.s. planes) are estimated using the full covariance matrix. The cell e.s.d.'s are taken into account individually in the estimation of e.s.d.'s in distances, angles and torsion angles; correlations between e.s.d.'s in cell parameters are only used when they are defined by crystal symmetry. An approximate (isotropic) treatment of cell e.s.d.'s is used for estimating e.s.d.'s involving l.s. planes.
Refinement. Refinement of *F*^2^ against ALL reflections. The weighted *R*-factor *wR* and goodness of fit *S* are based on *F*^2^, conventional *R*-factors *R* are based on *F*, with *F* set to zero for negative *F*^2^. The threshold expression of *F*^2^ > σ(*F*^2^) is used only for calculating *R*-factors(gt) *etc*. and is not relevant to the choice of reflections for refinement. *R*-factors based on *F*^2^ are statistically about twice as large as those based on *F*, and *R*- factors based on ALL data will be even larger.

### Fractional atomic coordinates and isotropic or equivalent isotropic displacement parameters (Å^2^)

**Table:**

	*x*	*y*	*z*	*U*_iso_*/*U*_eq_	
Br1	0.072488 (11)	0.38461 (3)	−0.022815 (15)	0.04538 (10)	
Br2	0.173873 (12)	0.97541 (3)	0.193709 (18)	0.04571 (10)	
Br3	0.038818 (9)	0.53557 (2)	0.168208 (16)	0.03360 (9)	
Br4	0.023492 (11)	−0.17162 (3)	0.074334 (14)	0.03793 (9)	
Cl1	0.21994 (3)	0.46769 (8)	0.54028 (4)	0.0503 (2)	
Cl2	0.19143 (3)	−0.20130 (9)	0.48243 (5)	0.0581 (2)	
O1	0.17220 (7)	0.23655 (17)	0.22437 (11)	0.0403 (5)	
O2	0.12506 (6)	0.26173 (17)	0.10327 (10)	0.0357 (4)	
H2O	0.1405	0.2323	0.1369	0.043*	
O3	0.22492 (6)	0.79766 (17)	0.28983 (10)	0.0356 (4)	
O4	0.06859 (7)	0.28669 (18)	0.41182 (10)	0.0393 (5)	
O5	0.05702 (6)	0.44299 (16)	0.31336 (10)	0.0303 (4)	
H5O	0.0593	0.4145	0.3504	0.036*	
O6	0.04818 (7)	−0.19587 (17)	0.22873 (10)	0.0372 (5)	
C1	0.15660 (8)	0.4293 (2)	0.17549 (13)	0.0258 (5)	
C2	0.13115 (8)	0.3794 (2)	0.11283 (14)	0.0288 (6)	
C3	0.10935 (8)	0.4552 (3)	0.05777 (13)	0.0309 (6)	
C4	0.11292 (8)	0.5753 (3)	0.06312 (14)	0.0320 (6)	
H4	0.0970	0.6244	0.0262	0.038*	
C5	0.14024 (8)	0.6289 (2)	0.12333 (13)	0.0277 (5)	
C6	0.14426 (9)	0.7552 (3)	0.12782 (14)	0.0322 (6)	
H6	0.1279	0.8037	0.0910	0.039*	
C7	0.17117 (9)	0.8070 (2)	0.18417 (15)	0.0308 (6)	
C8	0.19743 (8)	0.7369 (2)	0.23794 (14)	0.0285 (5)	
C9	0.19403 (8)	0.6138 (2)	0.23409 (13)	0.0268 (5)	
H9	0.2125	0.5664	0.2692	0.032*	
C10	0.16371 (8)	0.5568 (2)	0.17917 (13)	0.0251 (5)	
C11	0.17185 (8)	0.3462 (2)	0.23443 (14)	0.0292 (6)	
C12	0.18475 (8)	0.3844 (2)	0.30934 (13)	0.0265 (5)	
C13	0.22110 (9)	0.3333 (3)	0.35338 (15)	0.0345 (6)	
H13	0.2381	0.2788	0.3348	0.041*	
C14	0.23289 (9)	0.3612 (3)	0.42463 (15)	0.0384 (7)	
H14	0.2585	0.3295	0.4545	0.046*	
C15	0.20650 (9)	0.4361 (3)	0.45078 (14)	0.0328 (6)	
C16	0.16946 (9)	0.4855 (3)	0.40839 (15)	0.0328 (6)	
H16	0.1514	0.5353	0.4278	0.039*	
C17	0.15917 (8)	0.4610 (2)	0.33720 (14)	0.0288 (5)	
H17	0.1345	0.4967	0.3071	0.035*	
C18	0.24891 (9)	0.7304 (3)	0.34889 (15)	0.0358 (6)	
H18A	0.2668	0.7849	0.3832	0.043*	
H18B	0.2674	0.6724	0.3332	0.043*	
H18C	0.2290	0.6879	0.3707	0.043*	
C19	0.06148 (7)	0.2314 (2)	0.29361 (13)	0.0245 (5)	
C20	0.05402 (7)	0.3492 (2)	0.27021 (13)	0.0251 (5)	
C21	0.04405 (8)	0.3740 (2)	0.19710 (14)	0.0261 (5)	
C22	0.03838 (8)	0.2840 (2)	0.14929 (13)	0.0271 (5)	
H22	0.0319	0.3025	0.1007	0.033*	
C23	0.04202 (8)	0.1626 (2)	0.17117 (13)	0.0256 (5)	
C24	0.03339 (8)	0.0696 (2)	0.12090 (13)	0.0273 (5)	
H24	0.0256	0.0885	0.0723	0.033*	
C25	0.03620 (8)	−0.0467 (2)	0.14174 (13)	0.0275 (5)	
C26	0.04715 (8)	−0.0773 (2)	0.21398 (13)	0.0264 (5)	
C27	0.05499 (8)	0.0121 (2)	0.26305 (13)	0.0259 (5)	
H27	0.0614	−0.0085	0.3114	0.031*	
C28	0.05375 (7)	0.1344 (2)	0.24380 (13)	0.0231 (5)	
C29	0.07782 (8)	0.2141 (2)	0.37033 (13)	0.0276 (5)	
C30	0.10722 (8)	0.1132 (2)	0.39876 (13)	0.0273 (5)	
C31	0.14023 (8)	0.0807 (3)	0.36814 (13)	0.0296 (6)	
H31	0.1446	0.1245	0.3292	0.035*	
C32	0.16668 (9)	−0.0151 (3)	0.39424 (15)	0.0346 (6)	
H32	0.1893	−0.0377	0.3738	0.042*	
C33	0.15938 (9)	−0.0772 (3)	0.45092 (15)	0.0367 (6)	
C34	0.12720 (10)	−0.0470 (3)	0.48230 (15)	0.0391 (7)	
H34	0.1228	−0.0917	0.5209	0.047*	
C35	0.10132 (10)	0.0504 (3)	0.45627 (14)	0.0345 (6)	
H35	0.0794	0.0743	0.4780	0.041*	
C36	0.06237 (12)	−0.2311 (3)	0.30100 (15)	0.0438 (8)	
H36A	0.0619	−0.3185	0.3043	0.053*	
H36B	0.0431	−0.1969	0.3273	0.053*	
H36C	0.0917	−0.2020	0.3207	0.053*	

### Atomic displacement parameters (Å^2^)

**Table:**

	*U*^11^	*U*^22^	*U*^33^	*U*^12^	*U*^13^	*U*^23^
Br1	0.05153 (19)	0.0532 (2)	0.02754 (16)	−0.02078 (15)	0.00293 (13)	−0.00796 (13)
Br2	0.0600 (2)	0.01933 (17)	0.0526 (2)	−0.00021 (13)	0.00479 (16)	0.00278 (13)
Br3	0.04039 (16)	0.02086 (15)	0.04206 (17)	0.00226 (11)	0.01511 (13)	0.00746 (11)
Br4	0.05632 (19)	0.02872 (17)	0.02572 (15)	−0.00679 (13)	0.00481 (13)	−0.00455 (11)
Cl1	0.0497 (4)	0.0711 (6)	0.0289 (3)	−0.0060 (4)	0.0077 (3)	−0.0068 (3)
Cl2	0.0671 (5)	0.0552 (5)	0.0506 (5)	0.0325 (4)	0.0123 (4)	0.0199 (4)
O1	0.0628 (13)	0.0182 (10)	0.0390 (11)	0.0006 (9)	0.0116 (10)	−0.0024 (8)
O2	0.0475 (11)	0.0254 (10)	0.0342 (10)	−0.0079 (8)	0.0105 (9)	−0.0082 (8)
O3	0.0414 (11)	0.0228 (10)	0.0359 (10)	−0.0052 (8)	−0.0023 (8)	−0.0017 (8)
O4	0.0536 (12)	0.0319 (11)	0.0301 (10)	0.0092 (9)	0.0064 (9)	−0.0065 (8)
O5	0.0364 (10)	0.0221 (9)	0.0325 (9)	0.0025 (8)	0.0087 (8)	−0.0022 (7)
O6	0.0603 (13)	0.0202 (10)	0.0264 (10)	−0.0037 (9)	0.0025 (9)	0.0017 (7)
C1	0.0290 (12)	0.0217 (13)	0.0282 (13)	−0.0018 (10)	0.0103 (10)	−0.0019 (10)
C2	0.0323 (13)	0.0268 (14)	0.0307 (13)	−0.0056 (11)	0.0140 (11)	−0.0069 (11)
C3	0.0315 (13)	0.0377 (16)	0.0233 (12)	−0.0089 (11)	0.0062 (10)	−0.0056 (11)
C4	0.0316 (13)	0.0373 (16)	0.0258 (13)	−0.0028 (12)	0.0050 (11)	0.0014 (11)
C5	0.0297 (12)	0.0272 (14)	0.0263 (12)	−0.0013 (10)	0.0072 (10)	−0.0003 (10)
C6	0.0358 (14)	0.0268 (14)	0.0319 (14)	0.0016 (11)	0.0049 (11)	0.0058 (11)
C7	0.0375 (14)	0.0164 (13)	0.0382 (15)	−0.0007 (10)	0.0088 (12)	0.0012 (11)
C8	0.0302 (13)	0.0231 (13)	0.0307 (13)	−0.0021 (10)	0.0052 (10)	−0.0014 (10)
C9	0.0291 (12)	0.0227 (13)	0.0280 (13)	−0.0007 (10)	0.0063 (10)	0.0009 (10)
C10	0.0276 (12)	0.0227 (13)	0.0266 (12)	−0.0011 (10)	0.0100 (10)	−0.0009 (10)
C11	0.0320 (13)	0.0219 (14)	0.0353 (14)	−0.0027 (10)	0.0113 (11)	−0.0012 (11)
C12	0.0322 (13)	0.0197 (13)	0.0291 (13)	−0.0025 (10)	0.0103 (10)	0.0028 (10)
C13	0.0402 (15)	0.0302 (15)	0.0356 (15)	0.0088 (12)	0.0142 (12)	0.0044 (12)
C14	0.0360 (14)	0.0437 (18)	0.0345 (15)	0.0067 (13)	0.0074 (12)	0.0077 (13)
C15	0.0367 (14)	0.0371 (16)	0.0253 (13)	−0.0060 (12)	0.0095 (11)	−0.0009 (11)
C16	0.0331 (14)	0.0329 (15)	0.0354 (14)	0.0000 (11)	0.0140 (12)	−0.0044 (12)
C17	0.0268 (12)	0.0265 (14)	0.0333 (14)	0.0008 (10)	0.0080 (10)	0.0016 (11)
C18	0.0401 (15)	0.0295 (15)	0.0329 (14)	−0.0026 (12)	0.0003 (12)	−0.0015 (12)
C19	0.0213 (11)	0.0252 (13)	0.0265 (12)	0.0018 (9)	0.0050 (9)	0.0007 (10)
C20	0.0213 (11)	0.0226 (13)	0.0316 (13)	−0.0006 (9)	0.0069 (10)	−0.0015 (10)
C21	0.0256 (12)	0.0186 (12)	0.0347 (14)	0.0019 (9)	0.0086 (10)	0.0051 (10)
C22	0.0283 (12)	0.0271 (14)	0.0265 (12)	0.0012 (10)	0.0078 (10)	0.0051 (10)
C23	0.0251 (12)	0.0242 (13)	0.0267 (13)	−0.0001 (10)	0.0051 (10)	0.0025 (10)
C24	0.0323 (13)	0.0281 (14)	0.0203 (12)	−0.0018 (11)	0.0044 (10)	0.0007 (10)
C25	0.0331 (13)	0.0252 (14)	0.0224 (12)	−0.0043 (10)	0.0036 (10)	−0.0045 (10)
C26	0.0314 (12)	0.0211 (13)	0.0250 (12)	−0.0014 (10)	0.0037 (10)	0.0012 (10)
C27	0.0293 (12)	0.0241 (13)	0.0223 (12)	0.0000 (10)	0.0030 (10)	0.0024 (10)
C28	0.0221 (11)	0.0222 (13)	0.0244 (12)	0.0007 (9)	0.0047 (9)	−0.0003 (10)
C29	0.0310 (13)	0.0237 (13)	0.0266 (13)	−0.0019 (10)	0.0046 (10)	−0.0027 (10)
C30	0.0316 (13)	0.0261 (14)	0.0206 (12)	0.0000 (10)	−0.0002 (10)	−0.0031 (10)
C31	0.0278 (12)	0.0351 (15)	0.0242 (12)	−0.0025 (11)	0.0034 (10)	0.0039 (11)
C32	0.0283 (13)	0.0399 (16)	0.0334 (14)	0.0045 (12)	0.0037 (11)	0.0016 (12)
C33	0.0413 (15)	0.0332 (16)	0.0301 (14)	0.0108 (12)	−0.0008 (12)	0.0053 (12)
C34	0.0527 (17)	0.0389 (17)	0.0254 (13)	0.0080 (14)	0.0094 (12)	0.0071 (12)
C35	0.0442 (15)	0.0342 (16)	0.0259 (13)	0.0070 (13)	0.0108 (12)	0.0016 (11)
C36	0.071 (2)	0.0263 (15)	0.0273 (14)	0.0007 (14)	−0.0002 (14)	0.0052 (11)

### Geometric parameters (Å, °)

**Table:**

Br1—C3	1.896 (3)	C14—H14	0.9500
Br2—C7	1.892 (3)	C15—C16	1.384 (4)
Br3—C21	1.889 (2)	C16—C17	1.385 (4)
Br4—C25	1.899 (3)	C16—H16	0.9500
Cl1—C15	1.743 (3)	C17—H17	0.9500
Cl2—C33	1.745 (3)	C18—H18A	0.9800
O1—C11	1.243 (3)	C18—H18B	0.9800
O2—C2	1.336 (3)	C18—H18C	0.9800
O2—H2O	0.7916	C19—C20	1.396 (4)
O3—C8	1.353 (3)	C19—C28	1.441 (3)
O3—C18	1.435 (3)	C19—C29	1.482 (3)
O4—C29	1.240 (3)	C20—C21	1.422 (4)
O5—C20	1.338 (3)	C21—C22	1.359 (4)
O5—H5O	0.7825	C22—C23	1.419 (4)
O6—C26	1.356 (3)	C22—H22	0.9500
O6—C36	1.436 (3)	C23—C24	1.414 (4)
C1—C2	1.412 (4)	C23—C28	1.420 (3)
C1—C10	1.442 (4)	C24—C25	1.360 (4)
C1—C11	1.471 (4)	C24—H24	0.9500
C2—C3	1.415 (4)	C25—C26	1.419 (3)
C3—C4	1.350 (4)	C26—C27	1.369 (4)
C4—C5	1.417 (4)	C27—C28	1.417 (4)
C4—H4	0.9500	C27—H27	0.9500
C5—C10	1.417 (4)	C29—C30	1.487 (4)
C5—C6	1.419 (4)	C30—C35	1.386 (4)
C6—C7	1.353 (4)	C30—C31	1.394 (4)
C6—H6	0.9500	C31—C32	1.383 (4)
C7—C8	1.413 (4)	C31—H31	0.9500
C8—C9	1.381 (4)	C32—C33	1.385 (4)
C9—C10	1.411 (4)	C32—H32	0.9500
C9—H9	0.9500	C33—C34	1.374 (4)
C11—C12	1.491 (4)	C34—C35	1.387 (4)
C12—C13	1.389 (4)	C34—H34	0.9500
C12—C17	1.392 (4)	C35—H35	0.9500
C13—C14	1.394 (4)	C36—H36A	0.9800
C13—H13	0.9500	C36—H36B	0.9800
C14—C15	1.381 (4)	C36—H36C	0.9800
			
C2—O2—H2O	104.6	H18A—C18—H18C	109.5
C8—O3—C18	117.6 (2)	H18B—C18—H18C	109.5
C20—O5—H5O	104.4	C20—C19—C28	119.9 (2)
C26—O6—C36	117.7 (2)	C20—C19—C29	116.5 (2)
C2—C1—C10	118.9 (2)	C28—C19—C29	123.5 (2)
C2—C1—C11	116.4 (2)	O5—C20—C19	123.4 (2)
C10—C1—C11	124.6 (2)	O5—C20—C21	116.9 (2)
O2—C2—C1	123.0 (3)	C19—C20—C21	119.6 (2)
O2—C2—C3	117.1 (2)	C22—C21—C20	121.0 (2)
C1—C2—C3	119.9 (2)	C22—C21—Br3	120.9 (2)
C4—C3—C2	121.4 (2)	C20—C21—Br3	118.16 (19)
C4—C3—Br1	120.2 (2)	C21—C22—C23	120.7 (2)
C2—C3—Br1	118.4 (2)	C21—C22—H22	119.6
C3—C4—C5	120.6 (3)	C23—C22—H22	119.6
C3—C4—H4	119.7	C24—C23—C22	120.2 (2)
C5—C4—H4	119.7	C24—C23—C28	119.8 (2)
C4—C5—C10	120.3 (2)	C22—C23—C28	119.9 (2)
C4—C5—C6	120.0 (3)	C25—C24—C23	120.4 (2)
C10—C5—C6	119.7 (2)	C25—C24—H24	119.8
C7—C6—C5	120.4 (3)	C23—C24—H24	119.8
C7—C6—H6	119.8	C24—C25—C26	120.9 (2)
C5—C6—H6	119.8	C24—C25—Br4	120.40 (19)
C6—C7—C8	120.9 (2)	C26—C25—Br4	118.62 (19)
C6—C7—Br2	120.8 (2)	O6—C26—C27	124.9 (2)
C8—C7—Br2	118.3 (2)	O6—C26—C25	115.9 (2)
O3—C8—C9	124.7 (2)	C27—C26—C25	119.1 (2)
O3—C8—C7	116.0 (2)	C26—C27—C28	121.9 (2)
C9—C8—C7	119.2 (2)	C26—C27—H27	119.1
C8—C9—C10	121.4 (2)	C28—C27—H27	119.1
C8—C9—H9	119.3	C27—C28—C23	117.8 (2)
C10—C9—H9	119.3	C27—C28—C19	123.7 (2)
C9—C10—C5	117.8 (2)	C23—C28—C19	118.4 (2)
C9—C10—C1	123.7 (2)	O4—C29—C19	120.4 (2)
C5—C10—C1	118.5 (2)	O4—C29—C30	118.9 (2)
O1—C11—C1	120.8 (2)	C19—C29—C30	120.7 (2)
O1—C11—C12	115.4 (2)	C35—C30—C31	119.9 (2)
C1—C11—C12	123.7 (2)	C35—C30—C29	119.1 (2)
C13—C12—C17	119.5 (2)	C31—C30—C29	121.0 (2)
C13—C12—C11	118.7 (2)	C32—C31—C30	120.2 (3)
C17—C12—C11	121.5 (2)	C32—C31—H31	119.9
C12—C13—C14	120.5 (3)	C30—C31—H31	119.9
C12—C13—H13	119.7	C31—C32—C33	118.3 (3)
C14—C13—H13	119.7	C31—C32—H32	120.8
C15—C14—C13	118.4 (3)	C33—C32—H32	120.8
C15—C14—H14	120.8	C34—C33—C32	122.7 (3)
C13—C14—H14	120.8	C34—C33—Cl2	118.6 (2)
C14—C15—C16	122.1 (3)	C32—C33—Cl2	118.7 (2)
C14—C15—Cl1	118.9 (2)	C33—C34—C35	118.4 (3)
C16—C15—Cl1	119.0 (2)	C33—C34—H34	120.8
C15—C16—C17	118.7 (3)	C35—C34—H34	120.8
C15—C16—H16	120.6	C30—C35—C34	120.4 (3)
C17—C16—H16	120.6	C30—C35—H35	119.8
C16—C17—C12	120.6 (2)	C34—C35—H35	119.8
C16—C17—H17	119.7	O6—C36—H36A	109.5
C12—C17—H17	119.7	O6—C36—H36B	109.5
O3—C18—H18A	109.5	H36A—C36—H36B	109.5
O3—C18—H18B	109.5	O6—C36—H36C	109.5
H18A—C18—H18B	109.5	H36A—C36—H36C	109.5
O3—C18—H18C	109.5	H36B—C36—H36C	109.5
			
C10—C1—C2—O2	175.8 (2)	C28—C19—C20—O5	174.3 (2)
C11—C1—C2—O2	−7.9 (4)	C29—C19—C20—O5	−6.4 (3)
C10—C1—C2—C3	−6.8 (4)	C28—C19—C20—C21	−7.9 (3)
C11—C1—C2—C3	169.6 (2)	C29—C19—C20—C21	171.4 (2)
O2—C2—C3—C4	178.8 (2)	O5—C20—C21—C22	−176.9 (2)
C1—C2—C3—C4	1.2 (4)	C19—C20—C21—C22	5.1 (4)
O2—C2—C3—Br1	1.8 (3)	O5—C20—C21—Br3	3.0 (3)
C1—C2—C3—Br1	−175.81 (19)	C19—C20—C21—Br3	−174.94 (18)
C2—C3—C4—C5	2.7 (4)	C20—C21—C22—C23	0.4 (4)
Br1—C3—C4—C5	179.7 (2)	Br3—C21—C22—C23	−179.53 (19)
C3—C4—C5—C10	−0.9 (4)	C21—C22—C23—C24	176.0 (2)
C3—C4—C5—C6	179.0 (3)	C21—C22—C23—C28	−3.1 (4)
C4—C5—C6—C7	−178.1 (3)	C22—C23—C24—C25	−179.0 (2)
C10—C5—C6—C7	1.7 (4)	C28—C23—C24—C25	0.1 (4)
C5—C6—C7—C8	3.2 (4)	C23—C24—C25—C26	1.0 (4)
C5—C6—C7—Br2	−175.7 (2)	C23—C24—C25—Br4	178.73 (19)
C18—O3—C8—C9	−7.1 (4)	C36—O6—C26—C27	−6.0 (4)
C18—O3—C8—C7	173.8 (2)	C36—O6—C26—C25	175.3 (3)
C6—C7—C8—O3	176.3 (3)	C24—C25—C26—O6	178.7 (2)
Br2—C7—C8—O3	−4.7 (3)	Br4—C25—C26—O6	0.9 (3)
C6—C7—C8—C9	−2.9 (4)	C24—C25—C26—C27	−0.1 (4)
Br2—C7—C8—C9	176.1 (2)	Br4—C25—C26—C27	−177.86 (19)
O3—C8—C9—C10	178.3 (2)	O6—C26—C27—C28	179.4 (2)
C7—C8—C9—C10	−2.6 (4)	C25—C26—C27—C28	−1.9 (4)
C8—C9—C10—C5	7.3 (4)	C26—C27—C28—C23	2.9 (4)
C8—C9—C10—C1	−175.2 (2)	C26—C27—C28—C19	179.5 (2)
C4—C5—C10—C9	173.0 (2)	C24—C23—C28—C27	−2.0 (3)
C6—C5—C10—C9	−6.9 (4)	C22—C23—C28—C27	177.1 (2)
C4—C5—C10—C1	−4.6 (4)	C24—C23—C28—C19	−178.8 (2)
C6—C5—C10—C1	175.5 (2)	C22—C23—C28—C19	0.3 (3)
C2—C1—C10—C9	−169.1 (2)	C20—C19—C28—C27	−171.4 (2)
C11—C1—C10—C9	14.9 (4)	C29—C19—C28—C27	9.4 (4)
C2—C1—C10—C5	8.4 (4)	C20—C19—C28—C23	5.2 (3)
C11—C1—C10—C5	−167.6 (2)	C29—C19—C28—C23	−174.0 (2)
C2—C1—C11—O1	18.4 (4)	C20—C19—C29—O4	29.0 (4)
C10—C1—C11—O1	−165.5 (3)	C28—C19—C29—O4	−151.7 (3)
C2—C1—C11—C12	−157.1 (2)	C20—C19—C29—C30	−147.9 (2)
C10—C1—C11—C12	19.0 (4)	C28—C19—C29—C30	31.3 (4)
O1—C11—C12—C13	45.9 (4)	O4—C29—C30—C35	44.2 (4)
C1—C11—C12—C13	−138.4 (3)	C19—C29—C30—C35	−138.8 (3)
O1—C11—C12—C17	−128.2 (3)	O4—C29—C30—C31	−136.2 (3)
C1—C11—C12—C17	47.5 (4)	C19—C29—C30—C31	40.8 (4)
C17—C12—C13—C14	−2.2 (4)	C35—C30—C31—C32	1.0 (4)
C11—C12—C13—C14	−176.3 (3)	C29—C30—C31—C32	−178.5 (2)
C12—C13—C14—C15	3.1 (4)	C30—C31—C32—C33	0.2 (4)
C13—C14—C15—C16	−1.2 (5)	C31—C32—C33—C34	−0.4 (5)
C13—C14—C15—Cl1	177.9 (2)	C31—C32—C33—Cl2	177.9 (2)
C14—C15—C16—C17	−1.5 (4)	C32—C33—C34—C35	−0.6 (5)
Cl1—C15—C16—C17	179.4 (2)	Cl2—C33—C34—C35	−178.9 (2)
C15—C16—C17—C12	2.5 (4)	C31—C30—C35—C34	−2.0 (4)
C13—C12—C17—C16	−0.7 (4)	C29—C30—C35—C34	177.6 (3)
C11—C12—C17—C16	173.3 (2)	C33—C34—C35—C30	1.8 (5)

### Hydrogen-bond geometry (Å, °)

**Table:**

*D*—H···*A*	*D*—H	H···*A*	*D*···*A*	*D*—H···*A*
O2—H2O···O1	0.79	1.77	2.497 (3)	153
O5—H5O···O4	0.78	1.85	2.568 (3)	153
C4—H4···O4^i^	0.95	2.42	3.338 (3)	162
C18—H18A···Cl2^ii^	0.98	2.81	3.406 (3)	120
C34—H34···O2^iii^	0.95	2.49	3.397 (4)	160

Symmetry codes: (i) *x*, −*y*+1, *z*−1/2; (ii) −*x*+1/2, −*y*+1/2, −*z*+1; (iii) *x*, −*y*, *z*+1/2.
